# miR-21 enhances cardiac fibrotic remodeling and fibroblast proliferation via CADM1/STAT3 pathway

**DOI:** 10.1186/s12872-017-0520-7

**Published:** 2017-03-23

**Authors:** Wei Cao, Peng Shi, Jian-Jun Ge

**Affiliations:** 1 Department of Cardiology, The first Hospital of Anhui Medical University, Hefei, 230031 China; 2 Department of Cardiothoracic Surgery, The Second Hospital of Anhui Medical University, Hefei, 230601 China; 3 Department of Cardiology, The first Hospital of Anhui Medical University, Ji-Xi Road, Hefei, Anhui Province 230032 China

**Keywords:** Cardiac fibrosis, MicroRNA-21, CADM1, STAT3, Cardiac fibroblast

## Abstract

**Background:**

Cardiac fibrosis play a key role in the atrial fibrillation pathogenesis but the underlying potential molecular mechanism is still understood. However, potential mechanisms for miR-21 upregulation and its role in cardiac fibrosis remain unclear. The controls cell proliferation and processes fundamental to disease progression.

**Methods:**

In this study, immunohistochemistry, real-time RT-PCR, cell transfection, cell cycle, cell proliferation and Western blot were used, respectively.

**Results:**

Here we have been demonstrated that the tumor suppressor cell adhesion molecule 1 (CADM1) is the potential target of miR-21. Our study revealed that miR-21 regulation of CADM1 expression, which was decreased in cardiac fibroblasts and fibrosis tissue. The cardiac fibroblasts transfected with miR-21 mimic promoted miR-21 overexpression enhanced STAT3 expression and decreased CADM1 expression. Nevertheless, the cardiac fibroblasts transfected with miR-21 inhibitor obtained the opposite expression result. Furthermore, downexpression of miR-21 suppressed cardiac fibroblast proliferation.

**Conclusions:**

These results suggested that miR-21 overexpression promotes cardiac fibrosis via STAT3 signaling pathway by decrease CADM1 expression, indicating miR-21 as an important signaling molecule for cardiac fibrotic remodeling and AF.

## Background

Atrial fibrillation (AF) is considered to be an indication of underlying heart disease and is one of the most common cardiac arrhythmia disorders [1]. Cardiac remodeling, especially the structural remodeling, is a key result of AF [2]. Cardiac fibroblasts activation and aberrant fibroblast signaling can lead to cardiac fibrosis and heart injury [3]. Reports showed that cardiac remote ischaemic preconditioning can reduce periprocedural myocardial infarction in the percutaneous coronary interventions patients [4]. However, the molecular mechanisms of cardiac remodeling during atrial fibrillation remain incompletely understood.

MicroRNAs (miRs), small endogenous non-coding RNAs, about 19–22 nt functional RNA molecule, play important regulatory roles by sequence-specific base pairing on the 3' untranslated region (3'-UTR) of target messenger RNAs (mRNAs) [5]. miRs can post-transcriptionally silence protein expression either by binding to complementary target mRNAs and degrading these mRNAs, or by inhibiting mRNAs translating into proteins [6]. miRs are emerging as important regulators in participate in cell proliferation, differentiation, and apoptosis [7]. The effect of miR-21 on cardiac fibrosis has been well established [8, 9]. However, the precise function of miR-21 in cardiac fibrosis is still unclear.

Cell adhesion molecule 1 (CADM1), an immunoglobulin superfamily (Igsf) adhesion molecule, is a well-known tumor suppressor for a variety of cancers of epithelial origin [10]. CADM1 downregulation may through epigenetic silencing [11]. In immortalized kidney cells, the extracellular domain of CADM1 binds the receptor tyrosine kinase HER3, reducing cell proliferation [12]. CADM1 is representative cardio-miRs targets [13]. miRs regulate STAT3 signaling cascades that are involved in cardiac fibrosis as well as pathogenesis of human diseases [13]. STAT3 an important regulator of cell proliferation [14]. STAT3 is a downstream signaling molecule of CADM1 [15]. Currently, recent studies demonstrated that CADM1 suppress squamous cell carcinoma development through decreasing STAT3 activity [15].

This study was able to investigate the role of miR-21 and CADM1 in cardiac fibrosis and potential interaction between miR-21 and CADM1. More importantly, miR-21 inhibitor repressed cardiac fibrosis progression, miR-21 may be a better alternative to directly inhibit CADM1 expression in cardiac fibrosis. Our studies further support miR-21 acts as important regulators in participate in cardiac fibrosis pathological, enhances cardiac fibrotic remodeling and fibroblast proliferation via CADM1/STAT3 pathway.

## Methods

### Reagents and antibodies

Isoprenaline was obtained from He Feng (Shanghai, China). α-SMA and collagen I antibodies for were purchased from Boster (Wuhan, China), CADM1, p-STAT3 antibodies were obtained from Cell Signaling (Beverly, MA, USA). The primers of CADM1, STAT3, α-SMA, collagen I and β-actin were produced by Shanghai Sangong Biological and Technological Company (Shanghai, China). Immunohistochemical kit was obtained from the Zhongshan Biotechnology Corporation (Beijing, China). SYBR Green Real Master Mix and Reverse transcription reaction Mix were purchased from Fermentas (Ontario, Canada). Goat anti-rabbit IgG horse radish peroxidase (HRP), rabbit anti-goat IgG HRP, goat anti-mouse IgG HRP were obtained from Santa Cruz (California, USA).

### Animals and treatments

Sprague–Dawley (SD) rats (weight 180–230 g) were purchased from the Experimental Animal Center of Anhui Medical University. SD rats were randomly divided into vehicle group and model group. In the model group, we use the method of ISO (2.4 mg/kg/day, 7 days, *n* = 15) subcutaneous injection. The vehicle object treated the same procedure, and the only difference was that normal saline instead of ISO. After 1 week, heparin injection (625U/100 g), deep anesthesia was induced with pentobarbital (50 mg/100 g body weight), the rats were anesthetized and killed (the cervical dislocation: is the most common method of death in rat. With the thumb and index finger pressed down the rat head, the other hand to seize the tail, forced a little back above the pull, so that the cervical off, causing the spinal cord and brain marrow off, animals immediately died), hearts were quickly isolated and heart tissue specimens were fixed in 4% phosphate-buffered paraformaldehyde. Other heart tissue specimens were snap-frozen in liquid nitrogen and stored at –80 °C for molecular analysis.

### Patients and sample acquisition

All patients gave written informed consent to participate in this study, and the Institutional Ethics Committee of The First Hospital of Anhui Medical University (Anhui, China) approved the protocol. The study population consisted of 20 patients with severe isolated AF referred for mitral valve replacement in The First Hospital of Anhui Medical University. Twenty patients of SR underwent heart valve replacement surgery (Table 1). All selected patients were classified as heart function, based on the New York Heart Association (NYHA). During surgical replacement of the mitral valve myocardial samples were obtained from the left atrial appendage. Part of samples were immediately fixed in 10% buffered formalin and embedded in paraffin for immunohistochemical staining. The other samples were divided into pieces and frozen separately in liquid nitrogen following different processing for protein or RNA isolation.

**Table 1:** Clinical characteristics of all patients including of 20 SR patients and 20 AF patients

Group	SR	AF
Case (Male/Female)	20 (11/9)	20 (10/10)
Age (Year)	53.2 ± 10.5	46.7 ± 9.1
LVEF (%)	55.6 ± 6.9	56.8 ± 7.3
LAD (mm)	43.3 ± 4.2	58.1 ± 9.5**
NYHA-C (II/III/IV)	4/11/5	3/12/5

**Compared with SR group, *P* < 0.01

### Cell culture

The rat cardiac fibroblasts derive from SD neonate rats and cultured. A part of heart tissue was washed by PBS. Equal volumes of 0.25% trypsin and 0.3% type I collagen enzyme were added, and heart tissue slices were incubated in a 37 °C water bath. Cells were collected and the collected cells were filtered with a screen stencil and centrifuged at 1200 rpm for 5 min. Cells were seeded at a concentration of 2 × 10^4^ cells/cm^2^, and cultured with DMEM containing 10% (v/v) fetal bovine serum, supplemented with 100U/ml penicillin, 100 mg/ml streptomycin, 2 mM L-Glutamine, respectively. After four passages, cells can be cultured with 5 ng/ml recombinant murine TGF-β1 (Peprotech, USA). Cells were passaged for 48 h and serum- starved with DMEM for 24 h. Cell cultures were maintained at 37 °C in an atmosphere of 5% CO_2_.

### Histological analysis

The rats were anesthetized and killed, hearts were quickly isolated and the heart tissue fixed in 10% buffered formalin. The left atrial appendage of the patients done the same procedure. The heart tissue was excised. Tissues were embedded in paraffin and cut into 5 μm thick sections. They were mounted on normal glass slides and stained with hematoxylin & eosin (H&E) and Masson trichrome staining. Images of H&E-stained sections were used to measure cardiomyocyte width using Olympus Image Pro-plus. Each section was assessed under light microscopic fields.

### Immunohistochemistry

The tissue sections were formalin fixed and paraffin embedded prior to immunohistochemical staining. Sections were deparaffinized, rehydrated, processed for antigen retrieval. Sections were then incubated with primary antibodies against α-SMA (1:400), CADM1 (1:300) and p-STAT3 (1:200) overnight at 4 °C. After rinsing, the sections were subsequently incubated with a biotinylated goat anti-rabbit secondary antibody for 60 min at room temperature, washed in PBS, and stained with DAB(3, 3′-diaminobenzidine). Slides were counterstained with hematoxylin before dehydration and mounting. Negative controls were run in parallel by replacing the primary antibodies with PBS.

### miR-21 mimic and inhibitor transfection

miR-21 mimic, inhibitor and their respective negative control oligonucleotides were designed and chemically synthesized by Biomics Biotechnologies (Nantong, China). The sequences: miR-21 mimic sense 5′-UAGCUUAUCAGACUGAUGU UGA -3′and anti-sense 5′-UCAACAUCAGUCUGAUAAGCUA-3′, miR-21 inhibitor sequences were 5′-UCAACAUCAGUCUGAUAAGCUA-3′. Cardiac fibroblasts was prepared 24 h in antibiotic-free medium before transfection and cultured into six-well plate to achieve >70% confluence on the day of transfection. The miR-21 mimic, inhibitor or their respective NC was transfected into cells using Lipofectamine™ 2000 Reagent (Invitrogen, Carlsbad, CA, USA) in serum-free conditions for 4–6 h before changing to complete medium. The transfected cells were cultured in regular culture medium, and RNA and protein were collected 48–72 h after transfection, respectively.

### One-step qRT-PCR

To detect the expression of miR-21 in human atrial tissues, rat heart tissues and cardiac fibroblasts, qRT-PCR was carried out using the ABI 7500 Real Time PCR system (Foster City, CA, USA). Total RNAs were extracted through one-step method with Trizol (Invitrogen, USA). RNA-concentration was determined by IMPLEN NanoPhotometer. One-step real-time qPCR was performed to confirm expression of miR-21, it was measured using EzOmics miRNA qPCR Detection Primer Set (Biomics, USA) and EzOmics One-Step qPCR Kit (Biomics, USA). PCR was performed at 42 °C for 30 min; 95 °C for 10 min, followed by 40 cycles of amplification. Analysis the miR-21 expression was performed using the comparative CT method with U6 as endogenous controls, and the test was repeated three times.

### qRT-PCR

Total RNA was isolated from cells and tissues, and the first-strand cDNA was synthesized using RT-PCR synthesis kit according to the manufacturer's instructions. PCR for mRNA of CADM1 and STAT3 were performed by using RT-PCR kits. RT-PCR was carried out under standard protocol using the following primers (Table 2). PCR was performed at 42 °C for 30 min; 95 °C for 10 min, followed by 40 cycles of amplification. Analysis the miR-21 expression was performed using the comparative CT method with β-actin as endogenous controls, and the test was repeated three times.

**Table 2:** Primer sequences used for real time fluorescent quantitative polymerase chain reaction

Target mRNA	Primer	Sequence
Rat CADM1	Forward	5'-TGGCGTTACCTT GGTAACC-3'
	Reverse	5'-GGTGTTGAGCCCTTTCCAG-3'
Rat α-SMA	Forward	5' TGGCCACTGCTGCTTCCTCTTCTT 3'
	Reverse	5' GGGGCCAGCTTCGTCATACTCCT 3'
Rat STAT3	Forward	5'-CACCCATAGTGAGCCCTTGGA-3'
	Reverse	5'-TGAGTGCAGTGACCAGGACAGA-3'
Rat β-actin	Forward	5' TGG AATCCTGTGGCATCCATGAAAC 3'
	Reverse	5' ACGCAGCTCAGT AACAGTCCG 3'
Human CADM1	Forward	5'-TCAACACGCCGTACTGTCTG -3'
	Reverse	5'-GTGGGAGGAGGGATAGTTGTG -3'
Human STAT3	Forward	5'- GAAGAATCCAACAACGGCAG-3'
	Reverse	5'- TCACAATCAGGGAAGCATCAC-3'
Human β-actin	Forward	5'-GCTCGTCGTCGACAACGGCTC-3'
	Reverse	5'-CAAACATGATCTGGGTCATCTTCTC-3'

### Cell proliferation assay

Cell proliferation was determined by the 3-(4, 5-dimethylthiazol-2-yl)-2, 4-diphenyl- tetrazolium bromide (MTT) assay based on the formation of formazan from MTT by metabolically active cells. The cells were seeded in 96-well culture plates at a density of 5 × 10^3^ cells per well and transfected with miR-21 inhibitor and negative control, respectively. Twenty four hours later, cell proliferation was assessed. MTT was added to the medium at a final concentration of 5 mg/ml for 4–6 h. The resulting formazan crystals were dissolved in 150 μl dimethyl sulfoxide (DMSO), and measured at the optical density (OD) of 490 nm on a microplate reader (Bio-Tek EL, USA). All experiments were performed in triplicate and repeated at least three times.

### Cell cycle

Cell cycle analysis kit (Beyotime, China) for cell cycle analysis. The cells were seeded at a density of 5 × 10^3^ cells per well in 96-well culture plates and transfected with miR-21 inhibitor and their negative control, respectively. Cells were washed with cold PBS after fixation, then stained with 0.5 ml of propidium iodide (PI) staining buffer at 37 °C for 30 min in the dark. The cell cycle analyses were performed on BD LSR flow cytometer. The phases of cell cycle were analyzed by WinMDI software program. The experiments were performed in triplicate.

### Western blotting

Total protein samples were extracted, the protein concentration was detected using a BCA protein assay kit (Boster, China). Total protein from samples were electrophoresed on a 10% SDS-PAGE gel and blotted onto a PVDF membrane. After blocking, the membrane was probed with antibodies against CADM1, p-STAT3, α-SMA, collagen I and β-actin diluted in TBS/Tween20 (0.075%). Visualization of the second antibody was performed using a chemiluminescence detection procedure, ECL-chemiluminescent kit, according to the manufacturer’s protocol. The integrated density of each band was normalized to the corresponding β-actin band. β-actin was used as loading control.

### Statistical analysis

Experimental results are expressed as means ± SD. Statistical analysis was performed by using the ANOVA or the Student’s *t* test when appropriate. *P* value <0.05 was considered significant. All experiments were performed at least three times.

## Results

### Pathological changes

**Fig. 1:**
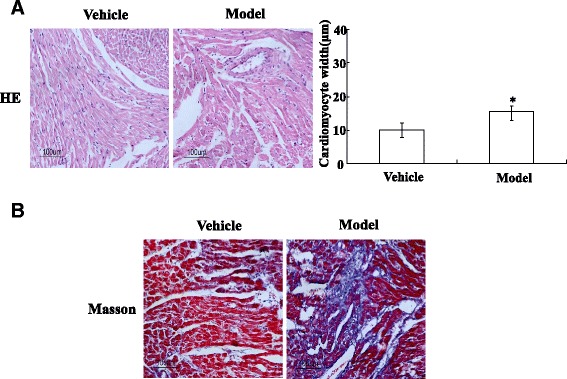
Pathological change in isoprenaline-caused rat cardiac fibrosis model. A piece of the heart tissue from each rat in the ISO rat model was fixed with formalin, and then it was embedded in paraffin. Thin sections were cut and stained with hematoxylin and eosin (H&E) (**a**), Masson’s trichrome stain (**b**). Rats were grouped vehicle (control) rat hearts or ISO (fibrotic) rat hearts. Representative views from each group are presented. The numbers in the views represent the groups of rats in the experiments

H&E staining revealed that the cardiomyocyte width was increased in the ISO group compared with the vehicle group (Fig. 1a) and Masson’s trichrome staining shown that cardiac collagen significantly deposition compared with the vehicle group (Fig. 1b). In sum, 1 week after ISO treatment result in cardiac fibrosis in rat heart.

### CADM1/STAT3 signal pathway activation in cardiac fibrotic remodeling and fibroblast

**Fig. 2:**
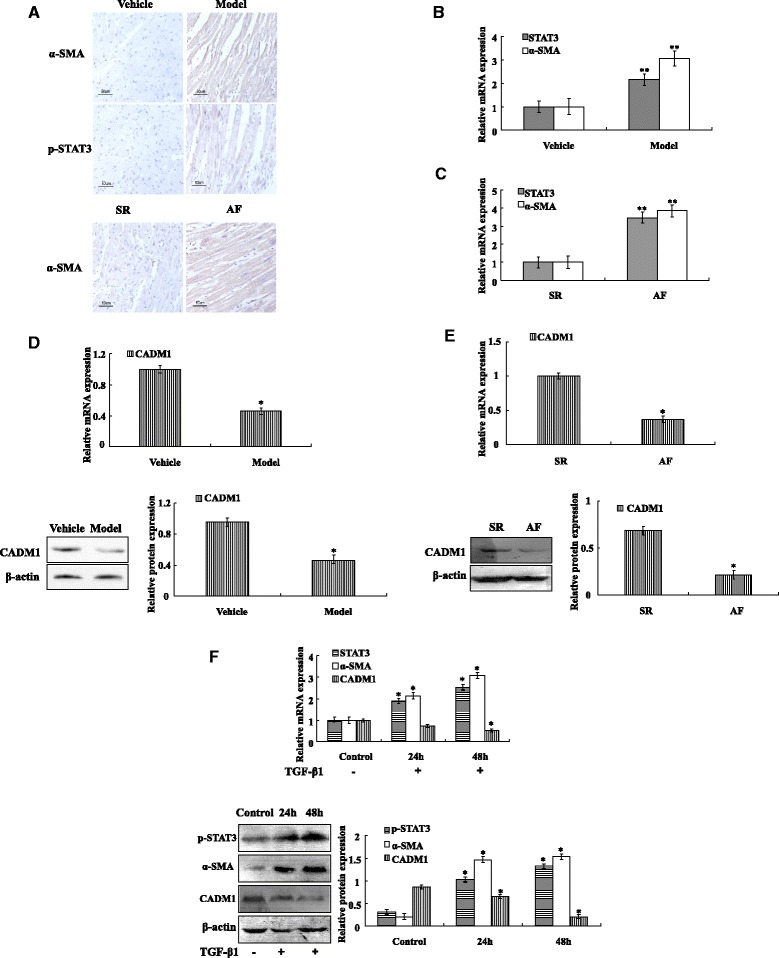
CADM1/STAT3 signal pathway activation in cardiac fibrotic remodeling and fibroblast. **a** p-STAT3 immunostaining on sections of vehicle rat heart or ISO (fibrotic) rat hearts. Images show absence of p-STAT3 in vehicle rat heart and selective p-STAT3 expression located in fibrotic lesions of diseased heart. Images show α-SMA staining localised selectively to smooth muscle cells lining vessels of vehicle rat heart and to myofibroblasts in association with fibrotic lesions in diseased heart. Moreover, Images show α-SMA staining localised selectively to smooth muscle cells lining vessels of SR heart and AF heart. **b** Rat heart tissues RNA was isolated, and STAT3, α-SMA expression were evaluated by qRT-PCR. **c** Human heart tissues RNA was isolated, and STAT3, α-SMA expression were evaluated by qRT-PCR. **d** Rat heart tissues RNA and protein was isolated, CADM1 mRNA expression was evaluated by qRT-PCR, CADM1 expression was analyzed by Western blotting. **e** Human heart tissues RNA and protein was isolated, CADM1 mRNA expression was evaluated by qRT-PCR, CADM1 expression was analyzed by Western blotting. **f** Total protein and RNA isolated from 0, 24 and 48 h cultures with TGF-β1 of rat CFs, STAT3, α-SMA and CADM1 expression were analyzed by Western blotting and qRT-PCR. Results are mean ± SD of triplicate experiments. *p < 0.05, **p < 0.01 vs control (vehicle)

Immunohistochemistry assay demonstrated that p-STAT3 and α-SMA protein expression were significantly increased in the model groups compared with the control (Fig. 2a). Moreover, immunohistochemistry assay found that α-SMA protein expression was significantly increased in the AF groups compared with the control (Fig. 2a). qRT-PCR suggested that STAT3 and α-SMA mRNA expressions in the rat model group and AF group were increased significantly compared with the control group (Fig. 2b; 2c). However, western blotting demonstrated that CADM1 protein expression was significantly decreased in the rat model group and AF group compared with the control group (Fig. 2d; 2e). qRT-PCR suggested that CADM1 mRNA expression in the rat model group and AF group were decreased significantly compared with the control group (Fig. 2d; 2e).

In vitro, western blotting and qRT-PCR shown that α-SMA and STAT3 expression increased in activated cardiac fibroblasts, which was treated with TGF-β1 compared with control group. However, western blotting and qRT-PCR found that CADM1 expression decreased in activated cardiac fibroblasts, which was treated with TGF-β1 compared with control group (Fig. 2f).

### miR-21 overexpression in cardiac fibrotic remodeling and fibroblast

**Fig. 3:**
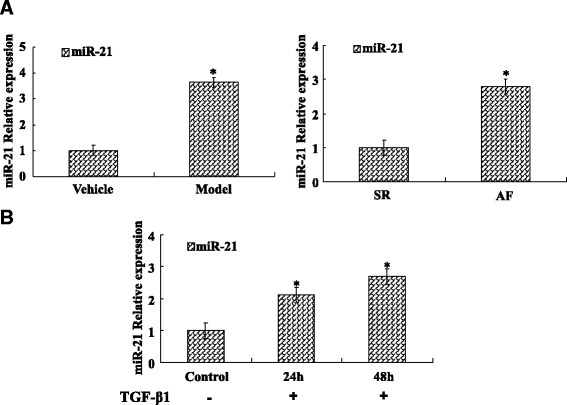
miR-21 increased in activated cardiac fibroblasts and fibrotic remodeling. **a** The miR-21 expression was assayed in rat and human cardiac fibrosis tissue. One Step Real-time PCR showed that miR-21 was significantly up-regulated in cardiac fibrosis tissue compared to vehicle tissue. Moreover, One Step Real-time PCR showed that miR-21 was significantly up-regulated in AF tissue compared to SR tissue. **b** The miR-21 expression was assayed in CFs stimulated with TGF-β1. One Step Real-time PCR showed that miR-21 was significantly increased in activated CFs compared to control group cell. Data are representative of at least three separate experiments. *P < 0.05 versus control or Vehicle or SR

To gain the expression of miR-21 in cardiac fibrotic remodeling and fibroblast. One-step qRT-PCR assay demonstrated that miR-21 expression in the rat model group and AF group were increased significantly compared with the control group (Fig. 3a). Moreover, One-step qRT-PCR found that miR-21 expression increased during activated cardiac fibroblasts, which was treated with TGF-β1 compared with control group (Fig. 3b).

### Knockdown of miR-21 inhibits the cardiac fibroblast proliferation

**Fig. 4:**
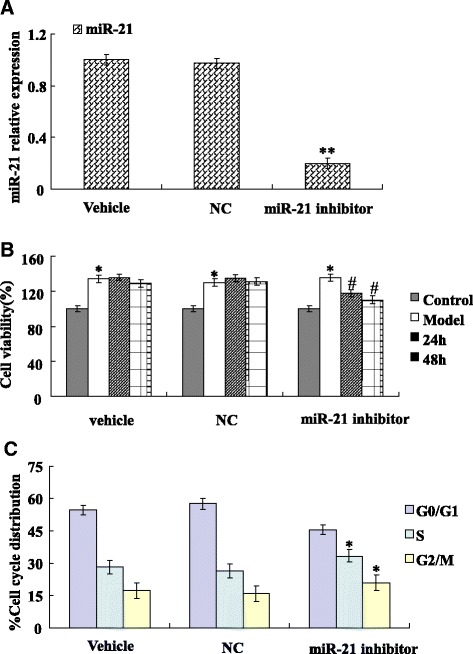
Knockdown of miR-21 inhibits the cardiac fibroblasts proliferation. **a** One Step Real-time PCR showed that transfection miR-21 inhibitor decreased the expression of miR-21 in cardiac fibroblasts. miR-21 inhibitor group is decreased significantly compare with vehicle group. **b** Cell viability was determined using MTT assay. 3 × 10^5^ cardiac fibroblasts cells were seeded in 96-well plates and transfected with 60 nM miR-21 inhibitor (24 h, 48 h). Knockdown of miR-21 caused a significant inhibition of cell activation and proliferation in TGF-β1-treated cardiac fibroblasts and the results were directly tested by MTT assay. **c** Cardiac fibroblasts cells were transfected with 60 nM miR-21 inhibitor for 48 h. Then cells were harvested and the cell-cycle distribution was analyzed by Flow cytometry analysis. Data are representative of at least three separate experiments. *P < 0.05, **P < 0.01 versus Vehicle or Control; ^#^ *P* < 0.05 versus model

To explore the roles of miR-21 in regulating cardiac fibroblasts cell proliferation, we tested the effect of miR-21 down-expression on the cell proliferation. By One-step qRT-PCR, we found that the expression of miR-21 significantly decreased in the cells transfected with miR-21 inhibitor compared with NC and vehicle (Fig. 4a). The cardiac fibroblast that was transfected with miR-21 inhibitor had a significantly lower proliferation than the NC and vehicle (Fig. 4b). Cell cycle distribution was analyzed by Flow cytometry analysis showed that anti-proliferative activity of miR-21 inhibitor was possibly due to induce G2/M phase arrest (Fig. 4c). These results suggested that down-expression of miR-21 inhibits the cardiac fibroblasts proliferation.

### miR-21 negative control CADM1 expression

**Fig. 5:**
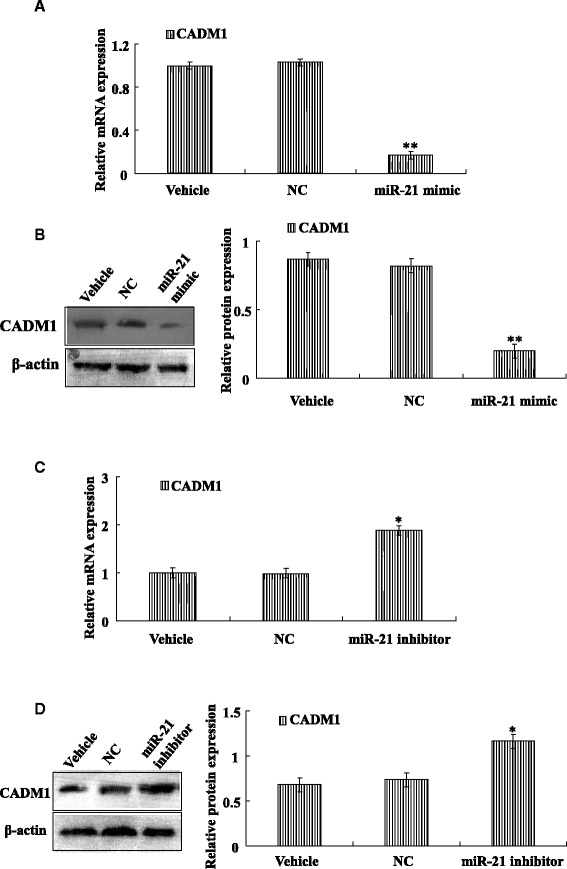
miR-21 negative control CADM1 expression. **a** Real-time PCR showed that transfection miR-21 mimic decreased the expression of CADM1 mRNA in cardiac fibroblasts cells. miR-21 mimic group is decreased significantly compare with vehicle group. **b** Western blotting indicated that transfection miR-21 mimic decreased the expression of CADM1 protein in cardiac fibroblasts cells. **c** Real-time PCR showed that transfection miR-21 inhibitor increased the expression of CADM1 mRNA in cardiac fibroblasts cells. miR-21 inhibitor group is increased significantly compare with vehicle group. **d** Western blotting indicated that transfection miR-21 inhibitor increased the expression of CADM1 protein in cardiac fibroblasts. Data are representative of at least three separate experiments. *P < 0.05, **P < 0.01 versus vehicle

To further determine the underlying mechanism of miR-21 during cardiac fibroblast activation. We began our studies interested in probing possible regulation of the CADM1 by miR networks in cardiac fibroblasts. To determine if miR-21 might play a key role in the dysregulation of the CADM1 expression. To explore the functional role of miR-21 on CADM1 targets, activated cardiac fibroblasts were transfected with miR-21mimics or a NC-miR for 48 h. RNA and protein were analyzed by PCR and Western blots, respectively. Interestingly, we found that CADM1 protein was significantly decreased compared with the NC and vehicle (Fig. 5b). Moreover, the mRNA level of CADM1 was significantly decreased compared with the NC and vehicle (Fig. 5a). However, knockdown of miR-21 with miR-21 inhibitor, PCR and Western blotting assay shown that the mRNA and protein level of CADM1 was significantly increased compared with the NC and vehicle (Fig. 5c; 5d). These results indicated that miR-21 as a potential regulator of CADM1 in cardiac fibroblasts.

### CADM1 inhibits STAT3 pathway activity

**Fig. 6:**
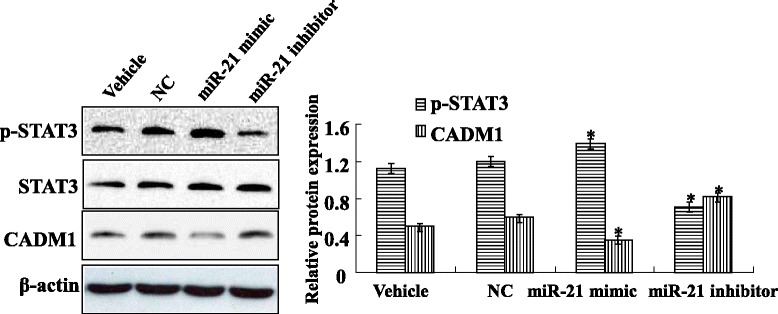
CADM1 inhibits STAT3 pathway activity. Western blotting indicated that transfection miR-21 mimic decreased the expression of CADM1 protein in cardiac fibroblasts cells. In addition, transfection miR-21 mimic increased the expression of p-STAT3 protein in cardiac fibroblasts cells. However, transfection miR-21 inhibitor increased the expression of CADM1 protein in cardiac fibroblasts cells. Moreover, transfection miR-21 inhibitor decreased the expression of p-STAT3 protein in cardiac fibroblasts cells. Data are representative of at least three separate experiments. *P < 0.05, **P < 0.01 versus vehicle

As mentioned above, miR-21 overexpression could enhance TGF-β1-induced cardiac fibroblasts activation by regulating CADM1/STAT3 signal in vitro, and then, activated cardiac fibroblasts were transfected with miR-21inhibitor (60nM) or a NC-miRNA (60 nM) for 48 h. The protein of CADM1 and STAT3 were detected by western blotting, compared to the NC and vehicle group, knockdown of miR-21 with miR-21 inhibitor, results shown that the protein level of CADM1 was significantly increased, and the protein level of p-STAT3 was significantly decreased (Fig. 6). Overexpression of miR-21 with miR-21 mimic, results shown that the protein level of CADM1 was significantly decreased, and the protein level of p-STAT3 was significantly increased (Fig. 6).

## Discussion

Cardiac fibrosis could appear as a result of AF [16]. Excessive fibroblast activation and collagen production are the major pathogenic mechanisms in the progression cardiac fibrosis [16, 17]. Therefore, the present study aimed to investigate the new mechanisms for cardiac fibroblast proliferation and cardiac fibrosis. Firstly, we evaluated the changes of pathological changes in the rat hearts. Masson’s and H&E assay shown that cardiac fibrosis rat collagen significantly deposition compared with the normal rat.

Abnormal expression of genes involved in cardiac fibroblast activation and fibrosis. Our study indicated that the mRNA and protein of STAT3 increased in activated fibroblast and fibrosis tissue. TGF-β1 induced fibroblasts activation and proliferation through STAT3. However, CADM1 mRNA and protein expression was significantly decreased in cardiac fibrosis tissue and fibroblast. Studies demonstrated that CADM1 may inhibit STAT3 activity and control cellular proliferation. Therefore, CADM1/STAT3 pathway is important for cardiac fibrosis development.

Emerging evidence suggests that miR-21 regulation of cardiac fibroblast activation and fibrosis [9, 18]. We demonstrated that the expression of miR-21 increased in cardiac fibroblast and fibrosis tissue. We also showed that silencing of miR-21 can inhibit α-SMA expression. Furthermore, the cell proliferation assay suggested that miR-21 inhibitors can suppress cardiac fibroblasts proliferation. However, it remains unclear whether miR-21 has an effect on CADM1 in cardiac fibroblast activation and fibrosis, we found that miR-21 overexpression correlated with CADM1/STAT3 in cardiac fibroblasts.

miRs therapy can control CADM1/STAT3 pathway, we examined what changes of CADM1/STAT3, after treatment cardiac fibroblasts with miR-21 mimics and inhibitors. Our experiment found that miR-21 may control CADM1 expression. Moreover, these data indicated that miR-21 suppress CADM1 may result in upregulation of STAT3 expression.

In sum, we found that miR-21 contribute to fibroblast proliferation and cardiac fibrosis via CADM1/STAT3 pathway. Our findings showed that miR-21 and CADM1 play a key role in cardiac fibrosis, indicating that miR-21, CADM1 and STAT3 may serve as therapeutic target of fibroblast activation and fibrosis. More importantly, we showed that miR-21 can inhibit cardiac fibroblasts proliferation, through regulation of the CADM1/STAT3 pathway, mediated by miR-21 and the targeting of the CADM1.

## Conclusions

Our findings indicated that miR-21 and CADM1 are involved in the pathogenesis of cardiac fibrosis, implicating that miR-21, CADM1 and STAT3 signaling pathway molecules could be used as therapeutic target of cardiac fibrosis. miR-21 can inhibit cardiac fibroblasts proliferation, through the CADM1/STAT3 pathway, mediated by miR-21, targeting CADM1.

## Abbreviation

AF: Atrial fibrillation
CADM1: Cell adhesion molecule 1
CVF: Collagen volume fraction
DMSO: Dimethyl sulfoxide
ISO: Isoprenaline
miRNA: MicroRNA
NC: Negative control
OD: Optical density
SR: Sinus rhythm
TGF-β1: Transforming growth factor beta 1
